# The impact on quality of life on families of children on an elimination diet for Non-immunoglobulin E mediated gastrointestinal food allergies

**DOI:** 10.1186/s40413-016-0139-7

**Published:** 2017-02-22

**Authors:** Rosan Meyer, Heather Godwin, Robert Dziubak, Julie A. Panepinto, Ru-Xin M. Foong, Mandy Bryon, Adriana Chebar Lozinsky, Kate Reeve, Neil Shah

**Affiliations:** 1 Department Paediatrics, Imperial College, London, UK; 2 Gastroenterology Department, Great Ormond Street Hospital for Children NHS Foundation Trust, London, UK; 3 Department of Pediatrics, Children’s Hospital of Wisconsin Research Institute/Medical College of Wisconsin, Hematology/Oncology/Bone Marrow Transplantation, Milwaukee, WI USA; 4 University College of London/Institute of Child Health, London, UK; 5 The Royal London Hospital, Paediatric A&E, Whitechapel Road, London, E1 1BB UK

**Keywords:** Non-IgE mediated allergies, Gastrointestinal allergies, Quality of life, Family impact score

## Abstract

**Background:**

The impact on health related quality of life (HRQL) has been well studied in children with Immunoglobulin E (IgE)-mediated food allergy. However limited data exists on related quality of life (QOL) of families who have a child suffering from food protein induced non-IgE mediated gastrointestinal allergies. We aimed to establish the QOL of families with children at the beginning of following an elimination diet for non-IgE mediated gastrointestinal food allergies.

**Methods:**

A prospective, observational study was performed. Parents of children aged 4 weeks–16 years who improved after 4–8 weeks of following an elimination diet for suspected non-IgE mediated allergies were included. The Family Impact Module (FIM) of the Pediatric Quality of Life (PedsQL™) was used and we compared our data to two historical cohorts: one with sickle cell disease and another with intestinal failure.

**Results:**

One hundred and twenty three children with a median age of 20 months were included (84 boys). The total FIM Score was 57.43 (SD 22.27) and particularly low for daily activities and worry. Factors that impacted significantly included age (*p* < 0.0001), number of foods excluded (*p* = 0.008), symptom severity (*p* = 0.041) and chronic nasal congestion (*p* = 0.012). Children with non-IgE mediated food allergies had worse scores in all domains (*p* < 0.0001) compared to sickle cell disease and worse physical (*p* = 0.04), emotional (*p* = 0.04) and worry (*p* = 0.01) domains compared to intestinal failure.

**Conclusions:**

This study found that parent QOL and family functioning was worse in those families who had a child on an elimination diet for non-IgE mediated allergies compared to those with sickle cell disease and intestinal failure, highlighting the impact this disease has on families.

## Background

The prevalence of food allergy in children ranges between 3 and 7%, with the majority of allergies caused by cow’s milk, hen’s egg, soya bean, wheat, peanut, tree nuts, fish and shellfish [1–3]. Current nomenclature classifies food allergies as either Immunoglobulin E (IgE) mediated, non-IgE or a mix of IgE and non-IgE mediated [4]. Food allergies affecting the gastrointestinal tract are mainly non-IgE mediated, but can also present with mixed symptoms. The most common reported symptoms include: diarrhoea, constipation, vomiting, severe abdominal pain, feeding difficulties and growth faltering [5]. In addition to the gastrointestinal symptoms, extra-intestinal manifestations including joint pain, lethargy and also severe sleep disturbances due to abdominal discomfort, are common [6]. The combination of aforementioned symptoms in addition to the burden and cost of the elimination diet may impact significantly on family life [7].

The impact on health related quality of life (HRQL) in children suffering from IgE-mediated food allergies and its effect on their families has been well studied [8–10].

Primeau et al. [11] indicated significantly more disruption in the familial/social dimension in families with a peanut allergic child, compared to parents of children with rheumatological disease. Similarly Sicherer et al. [12] also found an increase in emotional impact on parents and limitation on family activities in their cohort of IgE-mediated food allergic children. Particular mothers seem to be affected with higher levels of stress and anxiety [13]. In 2014 the European Academy for Allergy and Clinical Immunology (EAACI) published a position statement guiding the use of HRQL questionnaires in food allergic patients and their families [8]. The Food Allergy Quality of Life – Parent Burden questionnaire has been validated for IgE mediated, but not for non-IgE mediated allergies affecting the gastrointestinal tract [14]. The EAACI position statement highlighted the fact that there were only validated disease specific HRQL and family burden assessment tools for IgE-mediated food allergies and that there was a need for further studies to establish the burden of non-IgE mediated allergies and to develop new questionnaires. To date, the only data published on the family burden in non-IgE mediated allergies is the study by Klinnert et al. [7] on families with children with Eosinophilic Oesophagitis (EoE), a mixed non-IgE and IgE-mediated condition and Greenhawt et al. on Food Protein Induced Enterocolitis Syndrome (FPIES) [15]. Both studies indicated a significant impact on daily functioning of families in a variety of domains [7, 15]. Studies have also shown that having a child with chronic gastrointestinal pain or inflammatory bowel disease impacts significantly on family life [16, 17]. Limited data exists on the family impact of the whole spectrum of protein induced gastrointestinal non-IgE mediated allergies, which comprises a variety of diagnoses including proctocolitis, food protein enterocolitis syndrome, food protein induced dysmotlity, enteropathy and eosinophilic gastrointestinal disease. In our experience, it is in particular the initial period following diagnosis, where the burden is greatest on families. We therefore set out to establish the QOL of families with children at the beginning of following an elimination diet for non-IgE mediated gastrointestinal food allergies. We hypothesized that the families QoL would be much worse than families who have children with other chronic disease.

## Methods

### Subjects and procedure

A prospective, observational study was performed at the tertiary gastroenterology department, from Great Ormond Street Hospital for Children NHS Foundation Trust, London, United Kingdom (UK). Ethical approval was obtained for this study (Nr 11/LO/1177) from the NRES London-Bloomsbury Committee and only children with parental consent took part in this research project. Parents of children aged 4 weeks – 16 years without non-allergic co-morbidities (i.e., cerebral palsy, cardiac disorders) who were required to follow an elimination diet for the diagnosis of suspected non-IgE mediated food protein induced gastrointestinal allergies were approached for the study. The foods chosen for elimination was individualised, based on an allergy focused history performed by the gastroenterologist and also considered the success/failure of any previously eliminated foods. The elimination diet was managed by a specialist paediatric allergy dietitian, using the standard British Dietetic Association Diet sheets for specific food allergens. As we wanted to capture quality of life early at diagnosis, the inclusion in the study occurred if, after 4–8 weeks of following the elimination diet, there was an improvement in their gastrointestinal symptoms. This was measured by a repeated Likert scale gastrointestinal symptom questionnaire that has previously been published [18]. Children for this study were classified as non-IgE mediated allergy based on the success of the elimination diet and not on endoscopic evidence, as the majority did not undergo an endoscopic procedure, which is not routine practice in the UK. We also did not classify children into categories of food protein induced enteropathy, food protein induced enterocolitis syndrome, food allergic dysmotility or proctocolitis as in the first 4–8 weeks diagnosis is still established and the groups would be too small for statistical sub-analysis. We also collected data on atopic co-morbidities, including asthma, hayfever, atopic dermatitis and have included nasal congestion under this category, as our previously published work showed this a common complaint from parents [5].

We used the Family Impact Module of the Pediatric Quality of Life (PedsQl™) published by Varni et al. [19], that has been designed for use both as an adjunct to the existing PedsQL™ measurement scores or as a standalone assessment tool from 2 to 18 years of age. Permissions had been granted for the study to use this questionnaire from the Mapi research trust (http://www.mapi-trust.org/). The PedsQL Family Impact Module™ (FIM) [Version 2] questionnaire has been validated to measure impact for families with children ≥ 2 year of age. Although children in our allergic cohort were expected to be younger than 2 years of age, many previous studies have used the FIM in younger cohorts, including the publication by Sanchez et al. [20] that we used as historical control for this study [20–23]. In addition, using this module enabled us to compare our data to other cohorts. The FIM consists of 36 questions grouped into sections of Physical Functioning, Emotional Functioning, Social Functioning, Cognitive Functioning, Communication, Worry, Daily Activities and Family Relationships. Parents answered the questions on a 5 score Likert scale (0–4) where 0 means: “it is never a problem” and 4 means “it is almost always a problem”. Each score was then transformed into 0–100 scale in a following way: 4 = 0, 3 = 25, 2 = 50, 1 = 75, 0 = 100. If a parent scored more than half of questions in a section then an average score was calculated accounting for missing data. Average score of all questions represents Total Score. Parent QOL Summary Score is an average of scores from Physical, Emotional, Social, and Cognitive Functioning sections. Family Summary Score is an average of scores from Daily Activities and Family Relationships.

There are no cut-off values set for what is interpreted as “poor” QOL for families as reply to questions are subjective and depend on parental perception of their QOL. However, for the purpose of the study, all patients that had a PedsQL FIM™ score < 30 were contacted by the researchers and offered additional support.

Parents were sent the PedsQL FIM™ questionnaire and the research nurse assessed that all questions were completed at the time of the research appointment, which occurred 4–12 weeks after commencing the elimination diet. We compared our data to two historical control groups that fit the criteria of a chronic health related problem [22]. We obtained permission from Panepinto et al. [24] to use group mean scores from their PedsQL FIM™ from children with sickle cell disease (45.5% mild and 54.5% severe disease). Similarly, using the same questionnaire, we compared our results to those with intestinal failure (average bowel length 30 cm ±24) published by Sanchez et al. [20]. These two groups were chosen primarily because of available data using the same PedsQL FIM™ and because sickle cell disease represented a chronic disorder with milder symptoms whereas the children with intestinal failure were parenteral/enteral nutrition dependent with chronic symptoms and therefore embodied the severest end of spectrum of gastrointestinal disease.

### Statistical analysis

Descriptive statistics and logistic regression were done in IBM SPSS Statistics for Windows, Version 22 (Armonk, NY) and *t*-tests were done in R version 3.0.3 (R Foundation for Statistical Computing, Vienna, Austria). Two sided *t*-test was used to compare PedsQL FIM™ mean scores between food allergic group and sickle cell disease group. The same statistical technique was used to compare the intestinal failure to the allergic group, but we matched the ages and used only data of children < 7 years as Sanches et al. [20] only included children below this age (in three categories 1–12 months, 13–24 months and 2–6 year). A multiple linear regression model was used to assess the relationships between PedsQL FIM Total Score and number of foods excluded, the gastrointestinal symptoms score (taken from Likert scale questionnaires after 4–8 weeks from beginning of the elimination diet) and atopic features accounted for age (in years) and gender. Only variables which were significant were included in the model. Significance level was set to 0.05.

## Results

We identified 252 children with suspected non-IgE mediated gastrointestinal food allergies between December 2011 and November 2013 that were eligible for inclusion in the study. Ninety-one patients were excluded because they did not want to partake in the study, were unable to attend/not reachable or had non-atopic co-morbidities. Therefore 161 children were enrolled in the study, of which 30 patients did not improve on the elimination diet and consequently were excluded. Eight parents did not complete the PedsQL FIM™, therefore we analyzed questionnaires of 122 children. This cohort included 84 boys (68.3%) and the median age of the whole cohort was 20 months [IQR: 9 to 67.5]. Table 1 summarises the patient demographics, including the number and type of elimination diets implemented. The majority of children were on multiple exclusions, with milk, egg, wheat and soya being the most common combination. Gastrointestinal symptoms and the improvement of symptoms following the elimination diet have been reported in a previous publication [18]. In addition, 86% had at least one atopic co-morbidity, with persistent nasal congestion being the most commonly reported.

**Table 1:** Patient demographics

Variable	Data
Number of patients	123
Gender	84 boys (68.3%)
	38 girls (31.7%)
Age	20 month [IQR: 9 to 67.5]
Number of foods eliminated	
1	29 (23.6%)
2	33 (26.8%)
3	16 (13%)
4	23 (18.7%)
≥5	22 (17.9%)
Type of elimination^a^	
HF Only	14 (11.4%)
MES or MES+	11 (9%)
MEWS or MEWS+	33 (26.8%)
MS or MS+	30 (24.4%)
Other	15 (12.2%)
Single	20 (16.3%)
Atopic co-morbidities	
Eczema	60/122 (49%)
Asthma	36/122 (30%)
Allergic Rhinitis	25/122 (20%)
Nasal congestion	85/122 (70%)
At least one atopic feature	105/122 (86%)

^a^HF – hypoallergenic formula, MES – milk, egg soya, MEWS – milk, egg, soya, wheat, MS – milk, soya

In 111 (90.2%) of subjects, the mother filled in the questionnaire and 3 (2.4%) were completed by fathers. In 9 (7.3%) cases, parents did not indicate who was filling in the questionnaire. Due to the small number of fathers completing the questionnaire, we were not able to perform any statistical analysis on the differences in perceived QOL of the family between parents. The total average PedsQL FIM™ Score was 57.43 (SD 22.27) and while all domains indicated a poor QOL the impact of non-IgE mediated food allergies seemed to particularly affect daily family activities most and parents were very worried (Table 2).

**Table 2:** FIM in children with non-IgE mediated food allergy on QoL in different domains

	Number	Mean	Std. Deviation
Total Score	123	57.43	22.27
Parent QoL	123	58.95	23.25
Family Functioning	122	57.96	25.26
Physical Functioning	122	55.43	26.89
Emotional Functioning	123	55.72	24.13
Social Functioning	123	62.21	27.72
Cognitive Functioning	121	63.33	24.57
Communication	123	58.54	29.15
Worry	123	50.17	24.33
Daily Activities	122	48.84	31.84
Family Relationships	121	63.43	26.01

Multiple linear regression analysis was performed to ascertain the impact on families QOL (Total Average) of the following factors: gender, age, number of foods excluded, atopic co-morbidities (asthma, eczema and nasal congestion), persistent nasal congestion and gastrointestinal symptoms (individual and total score of symptoms) present after dietary elimination. The factors that impacted significantly (*p* < 0.05) on the families QoL included age (*p* < 0.0001), number of foods excluded (*p* = 0.008), total symptom score (*p* = 0.041) and nasal congestion (*p* = 0.012); implying that the higher number of foods excluded, the higher symptom severity, the younger age and the presence of nasal congestion negatively impacted QoL of families.

When comparing the family impact on QOL between our cohort and the patient group with sickle cell disease, we found that children with non-IgE mediated food allergies had worse scores in all domains. The difference was greatest in emotional and cognitive functioning and daily activities (Table 3). Similarly, when comparing our cohort to children with intestinal failure, we found that in all domains the mean score was lower in children with non-IgE mediated food allergy, but only the physical, emotional and worry domains achieved statistical difference (Table 4).

**Table 3:** QoL differences between children with Sickle Cell Disease and Non-IgE mediated allergy

	Sickle cell disease cohort		Non-IgE mediated Cohort		Mean difference	Statistical significance
	Mean	Std. Deviation	Mean	Std. Deviation	Between cohorts	*p* value
FIM Total Score	73.2768	18.39831	57.43	22.27	−15.8468	<0.0001
FIM Parent QOL	73.9996	18.70280	58.95	23.25	−15.0496	<0.0001
FIM Family Functioning	74.3541	22.25948	57.96	25.26	−16.3941	<0.0001
Physical Functioning	66.8246	22.96708	55.43	26.89	−11.3946	0.0011
Emotional Functioning	75.6744	20.96464	55.72	24.13	−19.9544	<0.0001
Social Functioning	80.2517	23.16745	62.21	27.72	−18.0417	<0.0001
Cognitive Functioning	76.0460	23.02157	63.33	24.57	−12.7160	0.0001
Communication	77.5709	23.72894	58.54	29.15	−19.0309	<0.0001
Worry	65.7270	26.32828	50.17	24.33	−15.5570	<0.0001
Daily Activities	69.7133	26.80064	48.84	31.84	−20.8733	<0.0001
Family Relationships	77.3271	23.67000	63.43	26.01	−13.8971	0.0001

**Table 4:** QoL differences between children with intestinal failure and non-IgE mediated allergy

	Intestinal failure		Non-IgE mediated cohort			Between cohorts	Statistical significance
	Mean	St Dev	*n*	Mean		mean difference	*p* value
TOTAL Average	61.3	17.32	100	53.92	21.86	−7.38	0.1332
Average QoL	63.48	15.64	100	55.07	22.58	−8.41	0.0931
Physical	62.34	17.83	99	50.56	25.79	−11.78	0.0401*
Emotional	64.13	19.23	100	53.05	23.98	−11.08	0.0409*
Social	60.05	26.96	100	57.96	27.63	−2.09	0.7431
Cognitive	66.96	21.78	100	60.13	24.26	−6.83	0.2176
Communication	64.49	26.97	100	54.08	28.17	−10.41	0.1099
Worry	61.74	21.25	100	47.91	24.3	−13.83	0.0132*
FAMILY Average	57.61	20.09	99	54.95	25.76	−2.66	0.6442
Daily Activities	46.38	29.92	99	44.02	31.36	−2.36	0.7436
Family Relationships	64.35	20.69	98	61.53	27.18	−2.82	0.6419

* *p* < 0.05

## Discussion

This study sets out to establish the impact of QOL on families with children who have non-IgE mediated food allergies affecting the gastrointestinal tract based on an elimination diet with symptoms improvement. The study aimed to capture the early impact of this food allergy on the QoL of families, who have often waited for months for a diagnosis.

In this study, we found that the average total PedsQL FIM™ score was worse than both intestinal failure and sickle cell disease. Our data indicates that families in our cohort are particularly affected in daily activities as well as physical and emotional functioning. Additionally the parents report significant worry about their child. Sources of stress for families of healthy children include the difficulties of feeding young babies, sleep issues, infant crying and social isolation [25–27]. Many of these aforementioned stresses are also experienced by parents of children with non-IgE mediated allergies, in addition to children struggling with sleep due to abdominal pain and feeding difficulties [28, 29]. The poor physical and emotional functioning of parents of children with non-IgE mediated allergies often starts with poor symptom recognition and delay in diagnosis [30], and when diagnosed, symptom management can often impose further stress. The mainstay for treatment for this allergy is an elimination diet that needs to be monitored and strictly adhered to. The fear of accidental exposure and therefore the burden of planning meals, sourcing free-from foods, ensuring that foods are not contaminated and often trusting others to keep to the dietary elimination, is significant [29]. Although this allergy is non-IgE mediated and parents do not need to worry about anaphylaxis, an inadvertent exposure to an offending allergen can lead to worsening of symptoms that can take a couple of days to weeks to improve [18]. Similar areas, affecting functioning of families of children with other chronic diseases, including diabetes and cancer have also been documented [22, 30].

Worry is a common emotion documented in particular in families of children with IgE mediated peanut allergy, due to the fear of their child dying [9, 11]. From our study, we do not know the exact reasons for the increased worry, but in EoE the worry is specifically related to medical management, problems with adherence to the diet, having diarrhoea/vomiting in front of other children and long term uncertain prognosis [31]. In the QoL study on FPIES, parents scored in particular high on concerns about nutrition, meal preparations, the child’s health and others not appreciating the severity of reactions [15]. In addition to this, we suspect that parents worry about the uncertain prognosis and often express their concern about their child deteriorating to where they were before the diagnosis/elimination diet and also the long term impact of medication used for management. In particular, mothers seem to be more affected when their child has a chronic disease. Greene [32] indicated that mothers of children with recurrent abdominal pain displayed greater anxiety, depression and somatization, which was also found in mothers of peanut allergic children [9]. Ninety-percent of our questionnaires were filled in by the mothers and we can therefore not rule out that perception of family impact is worse for the mothers than the fathers also in non-IgE mediated gastrointestinal allergies.

We have also found that the more foods that are eliminated the worse the impact on family QOL. This is not surprising at all, as the more foods are eliminated the more limited the commercial food choices are resulting in an increased need for home prepared foods. This also has a significant impact on social activities for both the child and the parent (i.e., what they can eat at a birthday party and eating out), in addition to the emotional well-being [31].

In 86% of our cohort, at least one co-morbidity (i.e., asthma, eczema, rhinitis and chronic nasal congestion) was present, which can impact on QOL irrespective of the presence of food allergy. Using regression analysis, we found that chronic nasal congestion was the only co-morbidity that significantly impacted on QOL. Eczema, hayfever and asthma, where present did not impact on the QoL of families. Interestingly nasal congestion has been found in 72% of children < 2 years of age with cow’s milk protein allergy in a study by Paddack et al. [33], which is similar to our findings. Nasal congestion affects both feeding coordination and sleep, both of which are important for maintaining the QOL of families.

The study results also indicated that it is not one symptom but the sum total of gastrointestinal symptoms (including the perceived severity) that impacts family QOL. Again this is not a surprising finding as studies on EoE have found QOL correlates with the severity of symptoms [7].

We hypothesised that the PedsQL FIM™ Score would be worse in our cohort than children with other chronic diseases [9, 10]. This hypothesis was proven correct in all domains of QOL when comparing our data to families of children with sickle cell disease. In the sickle cell cohort, 56% had severe sickle cell disease, but data on management was not available. Parents of children with this disease live in the fear of an acute sickle cell crisis, that may present with a vaso-occlusive or haemolytic crises affecting all organs and sepsis. However, patients with sickle cell disease also develop frequent and recurrent painful episodes throughout life, that worsen with age, thereby exhibiting chronic symptoms which are also seen in young children with non-IgE mediated gastrointestinal food allergies [18]. Nevertheless we suspect that the worse FIM scores of our non-IgE mediated cohort can be explained by the allergic group requiring a food elimination diet [12], sleep being affected with ongoing symptoms and the uncertain prognosis as non-IgE mediated gastrointestinal allergies remains poorly understood. Rouf et al. [29] found that mothers with food allergic children adjusted over time to develop strategies to overcome the emotional and practical challenges presented. However, in children with non-IgE mediated gastrointestinal allergies, the goal post for management and prognosis is constantly changing (i.e., can present with vomiting but then develop constipation later), thereby making it difficult to develop strategies to overcome emotional challenges [34].

We also compared our cohort to a group of children with intestinal failure aged between 6 months – 7 years (mean age 2.3 years) that are stable and dependant on either parenteral or enteral nutrition (56% had gastrostomies) [20]. The impact on families with artificial nutritional support is significant, as it entails frequent night-time feeding, concern about maintaining a sterile environment to prevent often life threatening infections and regularly requires procedures for re-inserting lines and changing gastrostomies. Similar to our cohort, this group would be dependent on optimal nutritional management to control symptoms and social and family life would be impaired due to the dependence on artificial nutrition. Although intestinal failure represents an extreme gastrointestinal condition, we were surprised to see that even compared to this group physical and emotional functioning, as well as worry about their child as significantly lower. Although there are no studies that explain this finding, we would hypothesise that this may be related to the knowledge of progression in disease with intestinal failure, where healthcare professionals understand the seriousness of the condition and there is a significant amount of research and support associations to help families, which is not yet the case for non-IgE mediated gastrointestinal allergies.

This study has several limitations. The first is related to the PedsQL FIM™ questionnaire itself; that it is not specific for families with children with non-IgE mediated food allergies [8, 35, 36]. Although the FIM has been used as means of assessing the impact on the family and measurement of self-reported functioning in the family, we are unable to assess the impact of the food allergy itself. The advantage however to using this generic tool is that we were able to compare this to other diseases [37–39]. In addition, the PedsQL FIM™ has been validated for children 2–18 years of age and we have used it in a younger cohort. Although this questionnaire has been used by many studies in children < 2 years of age, this limitation needs to be taken into account. It may also be perceived as a limitation of the study that inclusion of patients occurred based on symptom improvement following an elimination diet, rather than a formal challenge procedure. We wanted to establish QoL of family early on as this is when we have observed it to be the worst. All patients had subsequent home reintroductions of foods, however if we waited for these to occur, there would have been a delay of several months and QoL improves with time. Another limitation is the control groups, which were both historical and therefore not specifically selected as a control group for this study. In addition significant age differences exist between the sickle cell cohort and our patient group, which may have biased the data. The timing of completing the QOL questionnaire may also be a limitation. Parents completed the questionnaire 4–12 weeks after a successful elimination diet which may have affected scoring as coping skills often improve with time.

Strengths of this study include providing for the first time an indication of the burden for families of children with non-IgE mediated gastrointestinal allergies compared to other chronic diseases. It also highlights the specific domains of concern for families, namely worry, physical and emotional functioning. This knowledge can already impact on the advice and support given to families of children in the early stages of diagnosis when an elimination diet is initiated, when it is likely to impact most.

## Conclusion

This study has found that the QoL of parents and family functioning was worse in children on an elimination diet for non-IgE mediated gastrointestinal allergies compared to sickle cell disease and they also had worse scores in emotional and physical domains and worry than parents/families of children with intestinal failure. We found that the symptom severity, the number of foods avoided and also the presence of chronic nasal congestion impacts on QoL of families. We have highlighted with this study the impact this allergy has on families, which may help to inform clinical services with putting in place appropriate advice and support structures.

## Abbreviations

EoE: Eosinophilic Oesophagitis
FPIES: Food protein induced enterocolitis syndrome
HRQL: Health related quality of life
IgE: Immunoglobulin E
PedsQL FIM: Pediatric quality of life family impact module
QOL: Quality of Life
